# Biochar-mediated enhanced ethanol fermentation (BMEEF) in *Zymomonas mobilis* under furfural and acetic acid stress

**DOI:** 10.1186/s13068-020-1666-6

**Published:** 2020-02-26

**Authors:** Wei-ting Wang, Li-chun Dai, Bo Wu, Bu-fan Qi, Tian-fang Huang, Guo-quan Hu, Ming-xiong He

**Affiliations:** 1 Biomass Energy Technology Research Centre, Key Laboratory of Development and Application of Rural Renewable Energy (Ministry of Agriculture and Rural Affairs), Biogas Institute of Ministry of Agriculture and Rural Affairs, Section 4-13, Renmin Rd. South, Chengdu, 610041 People’s Republic of China; 2 Graduate School of Chinese Academy of Agricultural Science, Beijing, 100081 People’s Republic of China; 3 College of Pharmacy and Biological Engineering, Chengdu University, No. 2025, Cheng Luo Road, Chengdu, 610106 People’s Republic of China; 4 Chengdu Institute of Biology, Chinese Academy of Sciences, Section 4-9, Renmin Rd. South, Chengdu, 610041 People’s Republic of China

**Keywords:** Lignocellulosic hydrolysate, Furfural, Acetic acid, Biochar, *Zymomonas mobilis*

## Abstract

**Background:**

Pretreatment of lignocellulosic biomass generates different types of inhibitors (e.g., furfural and acetic acid), which could remarkably inhibit subsequent ethanol fermentation. Here, biochar as an additive in the fermentation broth was first applied to enhance ethanol production by *Z. mobilis* wild-type strain ZM4 in the presence of typical inhibitors.

**Results:**

This study showed that the biochar-mediated tolerance to furfural and acetic acid for the strain *Z. mobilis* ZM4 was the highest reported level, resulting in much higher ethanol productivity under stress conditions than that in non-treated conditions. Further analysis showed that adsorptive detoxification was not the controlling factor for enhanced ethanol production under stress conditions, attributed to its low removal of furfural (< 20%) and incapability of acetic acid removal. When biochar was filtered from the biochar-treated inhibitor-containing broth, it still showed enhanced ethanol production. Furthermore, *Z. mobilis* immobilized on biochar was also observed. Thus, biochar extracts in the fermentation broth and cell immobilization on biochar might be the controlling factors for enhanced ethanol production under stress conditions.

**Conclusions:**

These results indicate that biochar-mediated enhanced ethanol fermentation (BMEEF) might be a promising strategy for ethanol production from lignocellulosic biomass.
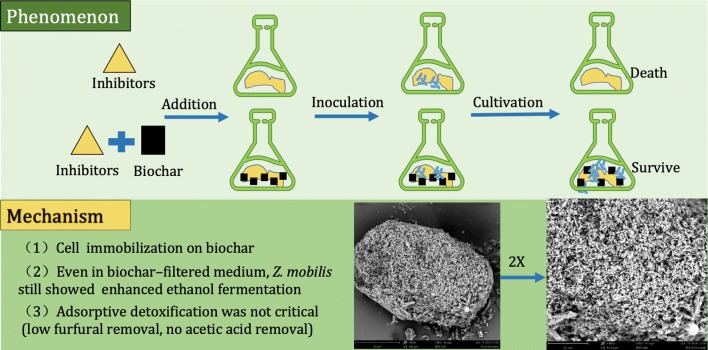

## Background

Cellulosic ethanol is a meaningful modern adjunct to fossil fuels and diluted acid pretreatment is one of the cost-effective methods reported and has been extensively studied [1]. However, the two most toxic inhibitors, i.e., furfural and acetic acid, were generated during this process, and these inhibitors would adversely affect the cellular growth, metabolism, and ethanol fermentation efficiency of ethanologenic bacteria [2, 3]. The minimum inhibitory tolerant levels vary from microbes but are generally below 1.5 g/L furfural and 3.0 g/L acetic acid, respectively [4, 5]. Furthermore, furfural can form synergistic inhibition with acetic acid [6]. Many methods that have been studied in the past focused on removing inhibitors before fermentation (e.g., physical, physicochemical and biological) [7, 8] or developing inhibitor-tolerant ethanologenic strains [9, 10].

*Zymomonas mobilis* is an excellent ethanologenic bacterium possessing several appealing characteristics such as high ethanol yield and tolerance capacity, low biomass amount, and high specific rate of sugar uptake [11, 12]. But its weak tolerance to furfural and acetic acid is the major drawback when applied to ethanol fermentation using lignocellulose feedstock containing furfural and acetic acid generated from pretreatment [13]. Apart from removing inhibitors before fermentation, creating mutants capable of tolerating furfural and acetic acid is efficient for the economic production of cellulosic biofuels [14, 15]. However, although several efforts have been applied to improve these inhibitors’ tolerance in *Z. mobilis*, including forward and reverse genetics, so far, the reported highest concentrations of furfural and acetic acid that *Z. mobilis* could tolerate were 3.0 g/L and 8.0 g/L [10, 16], respectively. But further efforts are still needed to meet the requirement of practical lignocellulose biorefinery with high inhibitor contents in the pretreated feedstock, and due to the complex mechanism of furfural and acetic acid stress in *Z. mobilis*, developing a robust strain will also be a difficult challenge [13–15].

Biochar, attributed to its special characteristics, such as high porosity, rich functional groups, abundant nutrients, is an emerging versatile material for various applications [17–23], such as soil amendment/remediation [19], crop production promotion [20], water pollution control [17, 18], and even anaerobic digestion [21, 22] and composting [23]. For example, in anaerobic digestion, biochar has shown its capacity in improving the fermentation of easy-acidification substrates by promoting buffering capacity [22]. However, little is known about the effects of biochar as a fermentation additive to improve ethanol fermentation by *Z. mobilis* in the presence of a high concentration of inhibitors in the hydrolysate [24, 25]. Biochar has shown its capacity in adsorptive detoxification of phenols, furfural, and 5-HMF [26–28]. However, biochar was not efficient in acetic acid adsorption. Moreover, adsorptive detoxification requires a high dose of biochar to achieve high removal of adsorbable inhibitors. A large amount of biochar for detoxification would impede its practical application. For example, the application of 4% biogas digestate-derived biochar in synthetic medium removed more than 94% of 5-HMF and 99% of furfural after 24 h of contact time [28].

Here, biochar-mediated enhanced ethanol fermentation (BMEEF) was developed by applying biochar as an additive in fermentation broth to promote ethanol production by *Z. mobilis* wild-type strain ZM4 in the presence of typical inhibitors. Adsorbable furfural and non-adsorbable acetic acid were chosen as typical inhibitors in this study. Biochar for BMEEF was not applied as a detoxification agent, so its dose was < 1/10 of the dose used for adsorptive detoxification [28], which was more suitable for practical application due to its much lower dose. Ethanol production under stress from furfural or acetic acid and co-stress was observed to confirm the effects of adsorptive detoxification, biochar extracts in the fermentation broth and immobilized *Z. mobilis* cells on biochar-enhanced ethanol production under various stress conditions. The results of this study could provide novel insights into the effects of biochar on ethanol fermentation under stress conditions.

## Results and discussion

### Biochar enhanced ethanol production under acetic acid stress

**Fig. 1:**
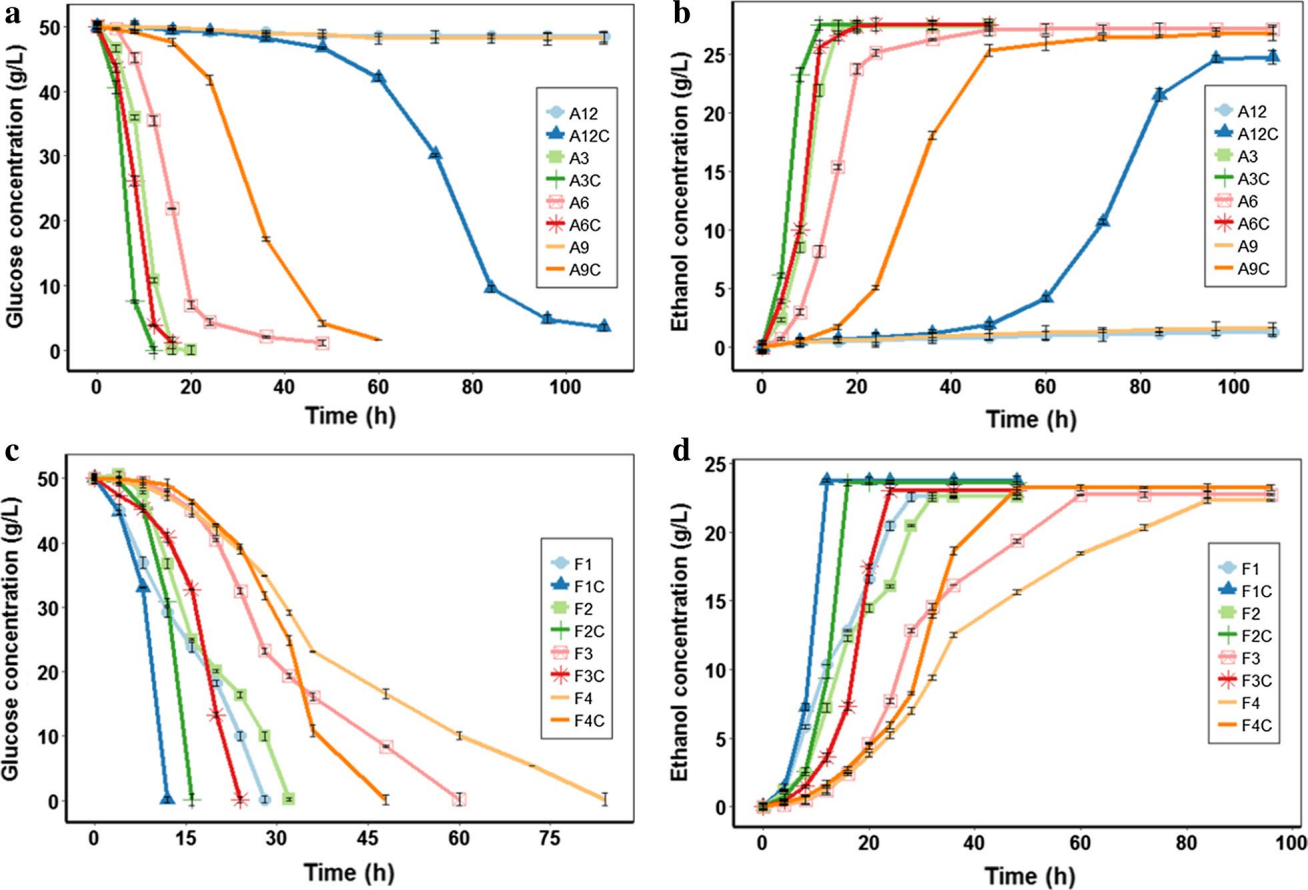
Effects of biochar on ethanol fermentation by *Z. mobilis* ZM4 in the presence of acetic acid stress condition (**a**, **b**), and furfural stress condition (**c**, **d**). Glucose indicates the concentration of sugar that remained in cultures. EtOH indicates the concentration of ethanol produced. “An” and “Fn” indicate *Z. mobilis* ZM4 fermented in the presence of *n* g/L acetic acid and *n* g/L furfural, respectively. “AnC” and “FnC” indicate *Z. mobilis* ZM4 co-cultured with 3.5‰ biochar fermented in the presence of *n* g/L acetic acid and *n* g/L furfural, respectively

Through forward and reverse genetics, the reported highest concentration of acetic acid that *Z. mobilis* could tolerate was 8.0 g/L [10]. Therefore, in this study, four different gradient concentrations (3.0, 6.0, 9.0, and 12.0 g/L) of acetic acid were employed. As shown in Fig. 1a, b, and Table 1, wild-type strain ZM4 was dramatically suppressed by 6.0 g/L acetic acid and could hardly survive under 9.0 g/L acetic acid. Actually, when a supplement of 6.0 g/L acetic acid was added, strain ZM4 consumed 97.86% glucose within 48 h, and when 9.0 g/L acetic acid was added, ZM4 could hardly survive, only consumed 6.24 ± 2% glucose within 96 h. However, with 3.5‰ biochar addition, strain ZM4 consumed 97.93% glucose and produced 25.10 ± 0.12 g/L ethanol within 16 h under 6.0 g/L acetic acid stress condition and consumed 92.0% glucose and produced 23.58 ± 1.03 g/L ethanol within 48 h with 9.0 g/L acetic acid addition. As reported, the highest concentration of acetic acid generated during the pretreatment step of biomass was nearly 10.0 g/L [29]. With 3.5‰ biochar addition, the ZM4 strain could consume 81.51% of the initial glucose after fermentation for 84 h in the presence of 12.0 g/L acetic acid. Considering that biochar was not efficient in acetic adsorption (Additional file 1: Figure S1), adsorptive detoxification might be not the controlling factor for enhanced ethanol production under acetic stress conditions. In anaerobic digestion conditions, biochar has also shown its capacity in improving the fermentation of easy-acidification substrates through its buffering capacity [22].

**Table 1:** Conversion of glucose to ethanol by *Z. mobilis* under acetic acid and/or furfural stresses

Strain	Fermentation time (h)	Glucose consumed (g/L)	Ethanol			Theoretical yield (%)	References
			Titer (g/L)	Yield (g/g glucose)	Productivity (g/L/h)		
50.0 g/L glucose + 3.0 g/L acetic acid							This study
ZM4 + C	12	52.60 ± 0.37	26.39 ± 0.18*	0.50 ± 0.00**	2.20 ± 0.02***	97.85	
ZM4	20	51.79 ± 0.58	24.83 ± 0.59	0.48 ± 0.01	1.24 ± 0.03	93.93	
50.0 g/L glucose + 6.0 g/L acetic acid							
ZM4 + C	16	51.47 ± 0.01***	25.10 ± 0.12***	0.49 ± 0.00***	1.57 ± 0.01***	95.89	
ZM4	48	50.61 ± 0.05	22.69 ± 0.08	0.45 ± 0.00	0.47 ± 0.00	88.06	
50.0 g/L glucose + 9.0 g/L acetic acid							
ZM4 + C	60	51.26 ± 0.00	23.58 ± 1.03***	0.46 ± 0.02***	0.39 ± 0.02	90.02	
ZM4	–	51.35 ± 0.18	0.18 ± 0.01	0.00 ± 0.00	–	–	
50.0 g/L glucose + 12.0 g/L acetic acid							
ZM4 + C	108	51.17 ± 0.10	21.79 ± 0.17***	0.43 ± 0.00***	0.20 ± 0.00	84.15	
ZM4	–	51.27 ± 0.04	0.06 ± 0.04	0.00 ± 0.00	–	–	
100.0 g/L glucose + 6.3 g/L acetic acid, pH 6.0							
ZM401	24	99.9	48.9	0.49	2.04	95.89	[30]
50.0 g/L glucose + 8.0 g/L acetic acid							
AQ8-1	64	49.59	20.76	0.42	0.32	82.19	[10]
AC8-9	72	41.85	21.46	0.43	0.30	84.15	
50.0 g/L glucose + 1.0 g/L furfural							This study
ZM4 + C	12	51.39 ± 0.02	23.77 ± 0.14**	0.46 ± 0.00**	1.98 ± 0.01***	90.02	
ZM4	28	51.39 ± 0.04	22.57 ± 0.23	0.44 ± 0.00	0.81 ± 0.01	86.11	
50.0 g/L glucose + 2.0 g/L furfural							
ZM4 + C	16	51.04 ± 0.47	23.57 ± 0.53	0.46 ± 0.01*	1.47 ± 0.03***	90.02	
ZM4	32	51.28 ± 0.56	22.21 ± 0.79	0.43 ± 0.01	0.69 ± 0.02	84.15	
50.0 g/L glucose + 3.0 g/L furfural							
ZM4 + C	24	51.42 ± 0.06	23.99 ± 0.88	0.47 ± 0.02	1.00 ± 0.04***	91.98	
ZM4	72	51.28 ± 0.19	22.74 ± 0.62	0.44 ± 0.01	0.32 ± 0.01	86.11	
50.0 g/L glucose + 4.0 g/L furfural							
ZM4 + C	48	51.18 ± 0.12**	22.82 ± 0.85*	0.45 ± 0.02*	0.48 ± 0.02***	88.06	
ZM4	84	51.64 ± 0.09	20.35 ± 1.09	0.39 ± 0.02	0.24 ± 0.01	76.32	
20.0 g/L glucose + 3.0 g/L furfural							
F211	22	N/A	N/A	0.46	N/A	90.02	[16]
F27	28	N/A	N/A	0.47	N/A	91.98	
20.0 g/L glucose + 3.0 g/L furfural							
ZM4-MF2	54	20	9.8	0.49	0.181	95.89	[31]

*P* values calculated by one-way ANOVA, * *P* < 0.05; ** *P* < 0.01; *** *P* < 0.001

Three repeats were performed for each fermentation

The BMEEF is an efficient and convenient method to promote the production of ethanol by *Z. mobilis* wild-type strain ZM4 under the high concentration of acetic acid. Compared with the reported acetic acid-tolerant *Z. mobilis* strains [4, 10, 30], biochar addition remarkably shortened the fermentation time and enhanced ethanol productivity. For example, a mutant ZMA7-2 (tolerant to 7.0 g/L acetic acid) was obtained via three rounds of adaptive laboratory evolution (ALE) [4], which consumed 96% glucose within 48 h. Besides, a flocculent mutant ZM401 and mutants ZMAQ8-1 and ZMAC8-9 with high tolerance to acetic acid were obtained by nitrosoguanidine (NTG) and ARTP mutagenesis, respectively [10, 30]. For ethanol fermentation by the acetic acid-tolerable mutant (ZM401), pH was maintained at 6.0 by KOH to promote ethanol production under 6.0 g/L acetic acid. Actually, when RM was supplemented with 6.0 g/L acetic acid, the pH value was 3.92. However, the pH value was uncontrolled for BMEEF. Moreover, although acetic acid-tolerable mutants (ZMAQ8-1 and ZMAC8-9) could produce ethanol with a high productivity at a certain stress condition, they were still not efficient in ethanol fermentation under high concentration of acetic acid (like in the presence of 8.0 g/L acetic acid), with the productivities of 0.32 and 0.3 g/L/h, respectively (Table 1) [10]. While in the presence of 9.0 g/L acetic acid, BMEEF by strain ZM4 could produce ethanol at a productivity of 0.39 ± 0.02 g/L/h. Thus, BMEEF was more efficient and convenient than the genetic engineering of strains.

### Biochar facilitated ethanol production under furfural stress

Furfural is another key inhibitor in the cellulosic hydrolysate. The highest concentration of furfural tolerated by *Z. mobilis* was 3.0 g/L in previous studies [16, 31]. Therefore, four different gradient concentrations (1.0, 2.0, 3.0, and 4.0 g/L) of furfural were employed in this study. As shown in Fig. 1c, d, and Table 1, *Z. mobilis* ZM4 was dramatically suppressed by over 2.0 g/L furfural. Actually, when a supplement of 3.0 g/L furfural was added, strain ZM4 consumed 99.64% glucose within 72 h. When 4.0 g/L furfural was added, strain ZM4 consumed 99.76% glucose within 84 h with a longer lag phase. However, with 3.5‰ biochar addition, strain ZM4 consumed 99.71% glucose and produced 23.99 ± 0.88 g/L ethanol within 24 h in the presence of 3.0 g/L furfural and consumed 99.72% glucose and produced 22.82 ± 0.85 g/L ethanol within 48 h under 4.0 g/L furfural stress. Thus, these results indicated that the addition of biochar remarkably reduced fermentation time under furfural stress conditions.

Various furfural-tolerant mutants were developed for ethanol production under furfural conditions. For example, mutant ZMF3-3 that is tolerant to 3.0 g/L furfural was screened through three rounds of ALE and consumed 80% glucose within 48 h [4]. In addition, mutant ZM4-MF2 was obtained by error-prone PCR of the global transcription sigma factor RpoD in *Z. mobilis* ZM4, which consumed 92.8% glucose and produced 9.8 g/L ethanol within 54 h in the presence of 3.0 g/L furfural under 20.0 g/L glucose [31]. However, with 3.5‰ biochar addition, strain ZM4 could consume 99.71% glucose and produce 23.99 ± 0.88 g/L ethanol within 24 h in the presence of 3.0 g/L furfural under 50.0 g/L glucose, which was more efficient in ethanol production under furfural stress condition than some of the previously reported inhibition-tolerant mutant. Thus, BMEEF was more efficient and convenient than the genetic engineering of strains.

### Biochar improved ethanol production under acetic acid and furfural stress

**Fig. 2:**
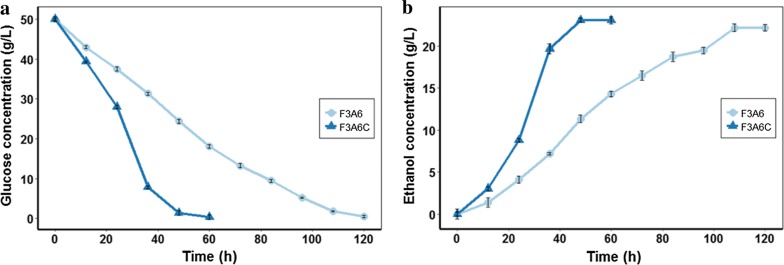
Effects of biochar on ethanol fermentation by *Z. mobilis* ZM4 in the presence of 3.0 g/L furfural plus 6.0 g/L acetic acid. **a** Glucose consumption. **b** Ethanol yield. F3A6 indicates *Z. mobilis* ZM4 fermented in the presence of 3.0 g/L furfural plus 6.0 g/L acetic acid. “F3A6C” indicates *Z. mobilis* ZM4 co-cultured with 3.5‰ biochar fermented in the presence of 3.0 g/L furfural plus 6.0 g/L acetic acid

Lignocellulosic hydrolysate is a complex system, and the high abundance of furfural can form synergistic inhibition with acetic acid [6]. Therefore, the BMEEF under co-stress by acetic acid and furfural was investigated. The concentrations of two inhibitors in a mixture were 6.0 and 3.0 g/L, respectively. As shown in Fig. 2a, b, strain ZM4 was dramatically suppressed by a mixture of these two inhibitors. While with 3.5‰ biochar addition, strain ZM4 consumed 97.15% of the initial glucose and produced 22.05 ± 0.58 g/L ethanol after fermentation 48 h, and in less than 60 h, strain ZM4 in untreated fermentation broth consumed 51.17% of the glucose. So far, there are few studies on multi-resistant *Z. mobilis* strains. The *Z. mobilis* mutant AcRIM0347 obtained by *hfq* gene insertion of *Z. mobilis* AcR was resistant to 0.75 g/L HMF, 1 g/L furfural or 1 g/L vanillin for 16, 19 or 21 h, respectively [9]. Although the mutant AcRIM0347 is resistant to a variety of inhibitors, the assay did not investigate a mixture of inhibitors. Thus, BMEEF might be a promising strategy to improve ethanol production under practical complex conditions with a variety of inhibitors.

### Mechanisms for BMEEF

#### Cell immobilization on biochar

**Fig. 3:**
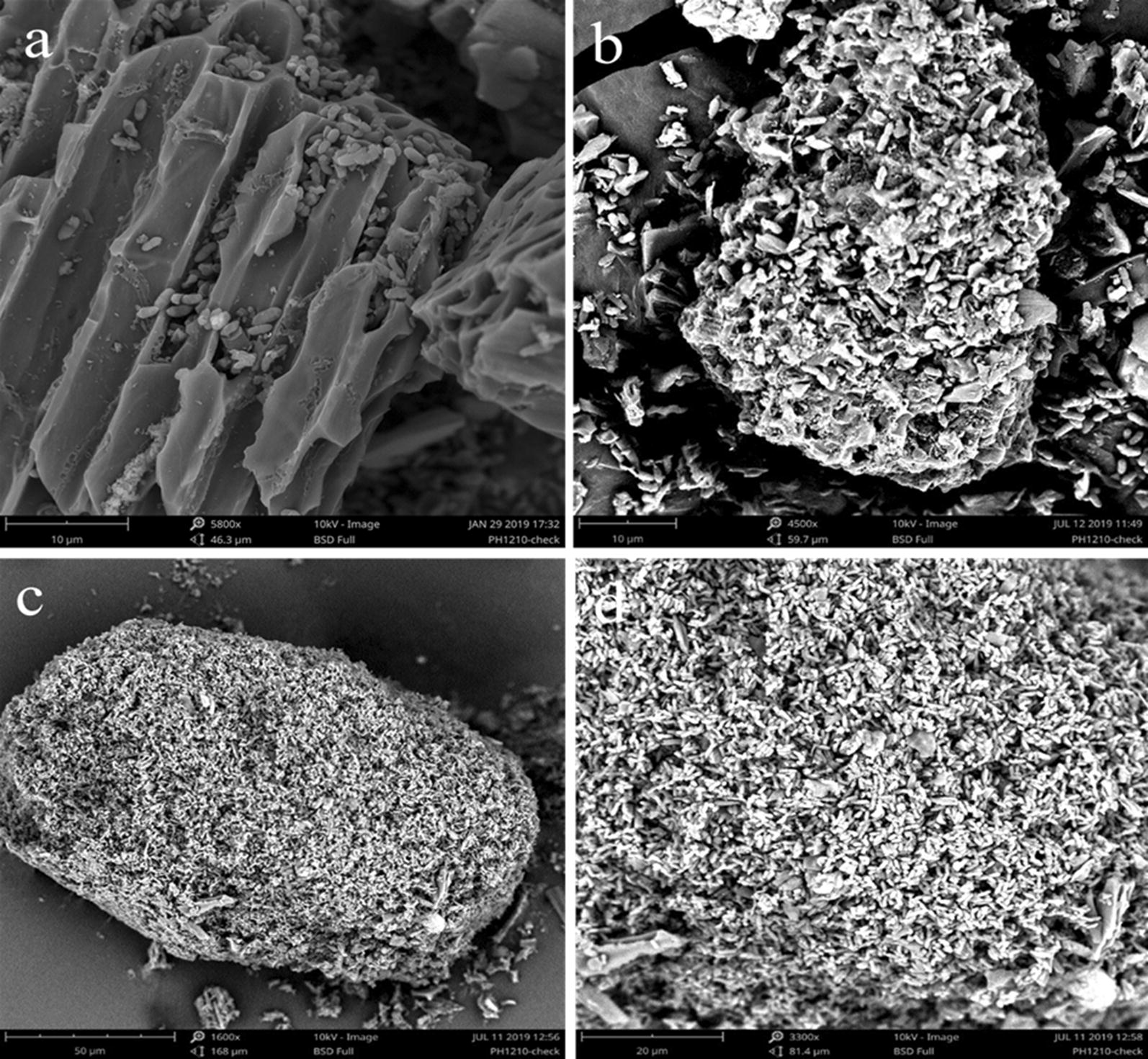
SEM of *Z. mobilis* interaction with biochar. **a** Biochar in RM medium with *Z. mobilis*. **b** Biochar in RM medium supplemented with 6.0 g/L acetic acid with *Z. mobilis.***c**, **d** Biochar in RM medium supplemented with 3.0 g/L furfural with *Z. mobilis*

The SEM images presented in Fig. 3 (biochar with *Z. mobilis* in RM medium, with *Z. mobilis* in RM medium supplemented with 6.0 g/L acetic acid, and with *Z. mobilis* in RM medium supplemented with 3.0 g/L furfural) highlighted the potential of biochar to provide a suitable habitat for microbial colonization. Cell immobilization on biochar in the fermentation broth and subsequent remarkable promotion of biofuel production were also observed by the ethanol fermentation by *S. cerevisiae* and *K. marxianus* in the presence of biowaste-derived biochar [25, 32]. However, the previous results about cell immobilization were obtained under conditions without inhibitors [33]. Here, the cell immobilization of *Z. mobilis* was observed under inhibition conditions, and, more importantly, immobilized *Z. mobilis* on biochar showed unexpected resistance to inhibitions. Thus, immobilizing *Z. mobilis* densely and homogeneously to the surface of biochar could be a novel strategy for bioethanol production, especially for bioethanol production under the practical condition with multi-inhibitors.

Two stages might be involved in the immobilization of microbes on biochar. The initial stage might be the adsorption of microbes onto biochar, which could be interpreted by the theory of colloid stability, and the second stage might be the biofilm formation [34, 35]. Cell immobilization on the surface of the biochar is a result of either physical adsorption by electrostatic force or due to natural cell attachment into the porous or covalent binding between the membrane and the support [36]. Further studies are needed to elucidate the formation of cell immobilization on biochar and the effects of immobilized ethanologenic strains on inhibition mitigation.

#### Unexpected effects of biochar extracts

**Fig. 4:**
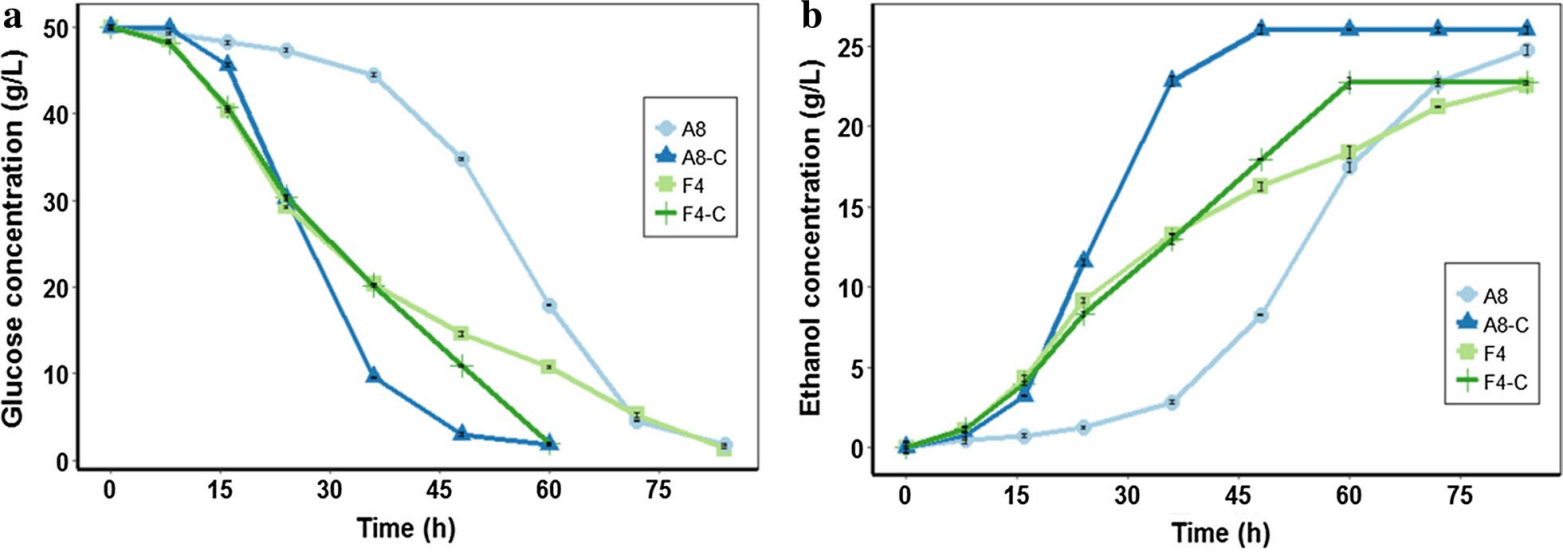
Effects of filtered medium of biochar on ethanol fermentation by *Z. mobilis* ZM4 “A8” and “F4” indicate *Z. mobilis* ZM4 fermented in the presence of 8.0 g/L acetic acid and 4.0 g/L furfural, respectively. “A8-C” and “F4-C” indicate *Z. mobilis* ZM4 co-cultured with filtered biochar-treated medium in the presence of 8.0 g/L acetic acid and 4.0 g/L furfural, respectively

Biochar in RM medium with inhibitor (8.0 g/L acetic acid or 4.0 g/L furfural) was filtered before strain inoculation to confirm the effects of biochar extracts on ethanol fermentation. As shown in Fig. 4, in the presence of 8.0 g/L acetic acid, strain ZM4 in the filtered biochar-treated medium showed a faster fermentation rate and consumed 93.98% glucose within 48 h, while strain ZM4 in biochar-untreated medium only consumed 30.44% glucose in less than 24 h. Moreover, in the presence of 4.0 g/L furfural, the fermentation time of ZM4 in the filtered biochar-treated medium was reduced by 24 h, compared to the biochar-untreated system. Thus, these results suggested that biochar extract promoted ethanol fermentation under stress conditions.

To further elucidate the effects of biochar extract, the nutrients in biochar-treated medium were investigated. Actually, 719.5 mg/L TN, 806 mg/L TOC, 674.2 mg/L TP, 193.2 mg/L K, and 12.03 mg/L Mg were detected in biochar-filtered medium, which were improved compared to raw RM medium. As previously reported, the glucose utilization of *Z. mobilis* is controlled by coupled phosphorylation; the addition of inorganic phosphate will lead to an improvement in the utilization of glucose by *Z. mobilis*, and Mg^2+^ ions, which has been confirmed to protect the viability of *Z. mobilis* via preventing the breakdown of RNA under starvation [37]. Besides, K^+^ ions are a co-factor of the most intracellular enzyme, and this property may benefit the growth of *Z. mobilis* and resistance to inhibitors. What’s more, some small molecules in biochar might act as allosteric regulators of a specific enzyme to facilitate resistance to inhibitors in the strain, and this needs further study. Thus, these results suggested that biochar could provide extra nutrients for promoting strain ZM4 resistant to acetic acid and furfural, facilitating ethanol production under stress conditions.

#### Beyond adsorptive detoxification

**Fig. 5:**
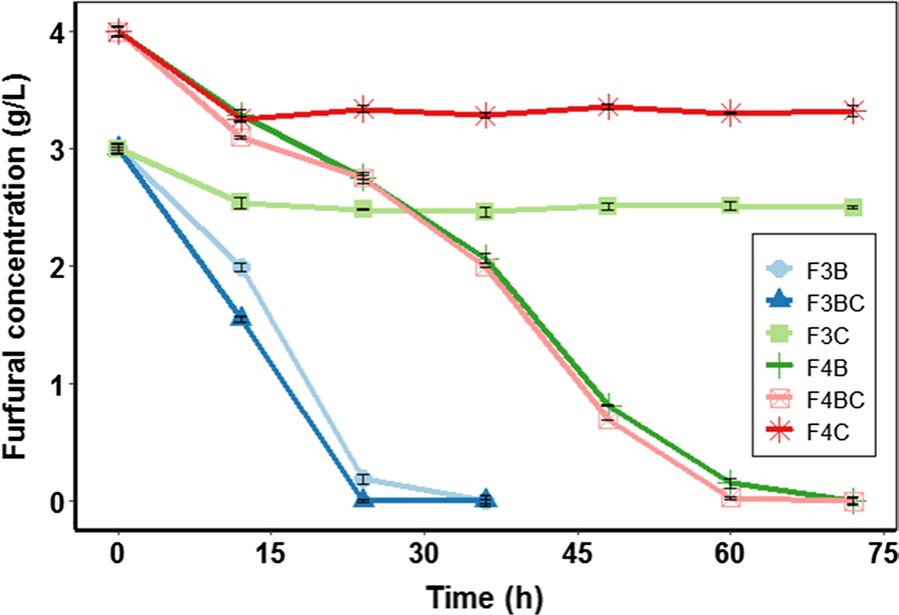
Effects of biochar on the adsorption of furfural. “FnB” indicates *Z. mobilis* ZM4 fermented in the presence of *n* g/L furfural. “FnBC” indicates *Z. mobilis* ZM4 co-cultured with 3.5‰ biochar fermented in the presence of *n* g/L furfural. “FnC” indicates biochar in the presence of *n* g/L furfural

Inhibitors such as furfural and HMF were prone to be adsorbed. However, a little adsorbent is highly efficient for acetic acid adsorption. Thus, adsorbable furfural and non-adsorbable acetic acid were chosen as two types of inhibitors in this study. As shown in Fig. 5, biochar adsorbed 15.25% and 18.75% of furfural in the presence of 3.0 and 4.0 g/L furfural within 12 h, respectively, indicating that the furfural concentration in the fermentation broth was still very high. In addition, the concentration of acetic acid was nearly invariable during the entire fermentation process (Additional file 1: Figure S1). Thus, these results suggested that adsorptive removal of inhibitors might not be the dominant mechanism for enhanced ethanol production under stress conditions.

Compared to traditional adsorptive detoxification [27], attributed to the low dose of biochar (3.5‰), the removal efficiency for furfural was lower in this study. For example, modified biochar from bamboo at a dose of 75 g/L was applied for furfural adsorption, and almost 100% furfural was removed in 10 g/L furfural solution [27]. However, for BMEEF, biochar was not used as an adsorbent for adsorptive detoxification, but as an additive. Thus, for BMEEF, strains were allowed to exist with a low dose of biochar and a certain amount of inhibitors in the fermentation broth.

Furthermore, compared to biochar application in other areas, e.g., soil amendment [19], anaerobic digestion [21, 22], and composting [23], attributed to its much lower application amount, the pH changes in BMEEF were not remarkable (Additional file 1: Figure S1). It only increased by 0.05 in all test conditions. Thus, pH adjustment was not responsible for enhanced ethanol production.

#### Biochar-mediated enhanced ethanol fermentation (BMEEF)

In this study, BMEEF was developed using 3.5‰ biochar as an additive to promote ethanol production, especially under stress conditions, not just as an adsorbent to remove inhibitors. Adsorptive removal of inhibitors by various adsorbents, e.g., biochar, activated carbon, has been developed for inhibition mitigation or detoxification. However, these adsorbents are not used as an additive in the fermentation broth, but used as a detoxification agent and removed out before ethanol fermentation [28]. When biochar was applied as a detoxification agent, it focused on the high removal of inhibitors. For example, previous results showed that more than 94% of 5-HMF and 99% of furfural were removed in the synthetic medium after 24 h of contact time by 4% biochar [28]. However, for BMEEF, the amount of biochar was as low as 3.5‰, which was much lower than the amount used in detoxification. Moreover, the removal of furfural by 3.5‰ biochar was < 20%, and no obvious decrease of acetic acid in the fermentation broth was observed. Thus, adsorptive detoxification was not critical for BMEEF, and strains were allowed to exist with a low dose of biochar and a certain amount of inhibitors.

Even under non-stress conditions, BMEEF could remarkably promote ethanol fermentation by *Z. mobilis*. As shown in Additional file 1: Figure S2, ethanol production in biochar-added RM medium without any inhibitors was investigated to confirm the effects of biochar on ethanol production. Results showed that, with the addition of 3.5‰ biochar in RM medium without any inhibitors, strain ZM4 consumed nearly 100% glucose and produced 27.44 g/L ethanol within 7.5 h, and in less than 4.5 h, strain ZM4 consumed 55.16% glucose and produced 15.09 g/L ethanol, indicating that biochar facilitated strain growth and subsequent ethanol production under non-stress conditions.

Mechanism analysis further suggested that BMEEF was not dependent on adsorptive detoxification due to its low removal for furfural and incapability in acetic acid removal in the fermentation broth, but dependent on the cell immobilization on biochar surface and biochar extract to supply nutrients (or some possible small organic molecules) to the broth. Further studies are needed to elucidate the formation process of cell immobilization on biochar, functional components in biochar extract, and their effects on strain growth and ethanol production under stress conditions.

Furthermore, attributed to the inhibition mitigation effects of BMEEF, it might be competent for the fermentation in concentrated hydrolysate with a high concentration of glucose and thus for the production of high concentration of ethanol in the fermentation broth, which needs further study. The immobilized strain on biochar might facilitate the recovery of strains in the fermentation broth, which could be beneficial for repeated batch fermentation [25, 32].

## Conclusion

In this study, the performance of BMEEF under various stress conditions (furfural and acetic acid) was first investigated. Results showed that with 3.5‰ (w/v) biochar in the fermentation broth, ethanol production was remarkably enhanced in the presence of high concentrations of furfural and acetic acid, especially under co-stress conditions. Further analysis showed that BMEEF was not dependent on adsorptive removal of inhibitors due to its low removal for furfural and incapability in acetic acid removal in the fermentation broth, but dependent on the cell immobilization on biochar surface and biochar extracts to supply nutrients (or some possible small organic molecules) to the broth. Thus, BMEEF might be a promising strategy for ethanol production from lignocellulosic biomass.

## Materials and methods

### Treatment of media and bacterial preparation

*Zymomonas mobilis* wild-type strain ZM4 was grown in rich medium (20.0 g/L glucose, 10.0 g/L yeast extract and 2.0 g/L KH_2_PO_4_) for the overnight stock culture. Biochar was prepared via the pyrolysis of wheat straw at 600 °C for 2 h at a heating rate of 10 °C/min in a vacuum tube furnace (MXQ1200-30, China) and then sieved to a particulate size of 80 mm [38].

Cultures were inoculated from a fresh plate of RM. The overnight stock culture was cultured for 12 h at 30 °C without shaking. The final optical density (OD) (Jingke UV765, Shanghai) of the overnight stock culture at 600 nm was 1.8. The OD_600_ was measured to assess the rate of bacterial growth. To 50 mL of RM medium, 3.5‰ (by weight, 0.175 g) biochar was added. Cell pellets were harvested from 2 mL of the overnight stock culture by centrifuging at 3000 rpm for 4 min at 4 °C. The harvested cell pellets were then inoculated into the two different media (RM and biochar-treated medium) to begin bacterial growth. In this set of experiments, bacteria grew in the presence of biochar or untreated RM medium throughout the time course (12 h) of each experiment. All growth and fermentations were carried out in triplicate.

An additional set of experiments was performed to investigate the influence of the biochar on typical inhibitors (adsorbable furfural and non-adsorbable acetic acid) generated during lignocellulose pretreatment. For these experiments, 3.5‰ (by weight, 0.175 g) biochar was added to 50 mL RM media (in 100 mL flask) with different acetic acid concentrations (3.0, 6.0, 9.0, and 12.0 g/L), or different furfural concentrations (1.0, 2.0, 3.0, and 4.0), or 6.0 g/L acetic acid plus 3.0 g/L furfural. Meanwhile, biochar-untreated RM medium was also supplemented with the corresponding concentrations of acetic acid or/and furfural as a control. 2 mL of the overnight stock culture was harvested and inoculated into each flask and placed in the incubator at 30 °C without shaking. All growth and fermentations were carried out in triplicate.

In addition, the third set of experiments was performed where biochar was removed from the medium before inoculation. For these experiments, the same 3.5‰ (by weight, 0.175 g) biochar was added to 50 mL RM media with 8.0 g/L acetic acid and 4.0 g/L furfural, respectively. Then the media was first filtered through a 0.45-μm sterile syringe filter and then passed through a 0.22-μm sterile syringe filter. Meanwhile, as a comparison, biochar-untreated RM medium was also supplemented with 8.0 g/L acetic acid and 4.0 g/L furfural, respectively. Cultivation methods were the same as the first and second sets of experiments. All cell growth and ethanol fermentation were carried out in triplicate.

### Characteristics of biochar

The mineral elements (e.g., K, Mg) present in the biochar were determined by ICP (PlasmaQuant PQ9000, Germany). The N content in the biochar was determined by a TN analyzer (SHIMADZU, Japan). The total carbon (TC) content and total organic carbon (TOC) contents were determined by a TOC-V CPH analyzer (SHIMADZU, Japan).

### SEM analysis

Biochar inoculated with *Z. mobilis* ZM4 cells was imaged using scanning electron microscope (SEM). Samples were prepared by chemical fixation and freeze-drying. Briefly, two milliliters of the co-cultures was first fixed with 2.5% glutaraldehyde at 4 °C for 12 h, rinsed by 0.1 M PBS buffer (pH 7.0) twice, dehydrated with graded ethanol, treated with isoamyl acetate (for replacement of ethanol), and then the samples were observed by scanning electron microscope (Phenom Pro, Netherlands Phenom) after freeze-drying.

### Analytical methods

After filtration with a 0.22-μm membrane and ten-time dilution, the concentrations of glucose, ethanol, acetic acid, and furfural in fermentation medium were analyzed by high-performance liquid chromatography (HPLC; Agilent 1200), equipped with refractive index detector. An HPX-87H ion exclusion column (Bio-Rad Aminex) was used at 65 °C with 5 mM H_2_SO_4_ as the mobile phase and run at a flow rate of 0.6 mL/min. The injection volume was set to 20.0 μL. One-way ANOVA was used to test all experimental data and the correlation was analyzed with the Pearson test (two tailed) using Statistical Product and Service Solutions Software (SPSS, version 19.0).

## Supplementary information

**Additional file 1: Figure S1.** Biochar-facilitated ethanol fermentation in acetic acid stress conditions. No acetic acid removal (a), and little pH changes (b). **Figure S2.** Effects of biochar on ethanol fermentation by *Z. mobilis* ZM4 in the presence of RM medium. “RM” indicates *Z. mobilis* ZM4 fermented in RM medium. “RMC” indicates *Z. mobilis* ZM4 co-cultured with 3.5‰ biochar fermented in RM medium.

## Data Availability

*Z. mobilis* AQ8-1 and AC8-9 have been deposited at Guangdong Microbial Culture Center (GDMCC) under the Accession number GDMCC60258 and 60259.

## Abbreviations

BMEEF: Biochar-mediated enhanced ethanol fermentation
ARTP: Atmospheric and room temperature plasma
ALE: Adaptive laboratory evolution
NTG: *N*-Methyl-*N*-nitro-*N*-nitrosoguanidine
RM: Rich medium
OD: Optical density
HPLC: High-performance liquid chromatography
SEM: Scanning electron microscope
