# Supplementary material for: Biochar-mediated enhanced ethanol fermentation (BMEEF) in Zymomonas mobilis under furfural and acetic acid stress
Source: Biotechnol Biofuels. 2020 Feb 26;13:28. doi: 10.1186/s13068-020-1666-6 (PMC7045489; doi:10.1186/s13068-020-1666-6)
Supplement: Supplementary file 1 — Additional file 1: Figure S1. Biochar-facilitated ethanol fermentation in acetic acid stress conditions. No acetic acid removal (a), and little pH changes (b). Figure S2. Effects of biochar on ethanol fermentation by Z. mobilis ZM4 in the presence of RM medium. “RM” indicates Z. mobilis ZM4 fermented in RM medium. “RMC” indicates Z. mobilis ZM4 co-cultured with 3.5‰ biochar fermented in RM medium. [file 13068_2020_1666_MOESM1_ESM.docx]

Additional file 1

**Biochar-mediated Enhanced Ethanol Fermentation (BMEEF) in *Zymomonas mobilis* Under Furfural and Acetic Acid Stress**

Wei-ting Wang^1, 2†^, Li-chun Dai^1†^, Bo Wu^1^, Bu-fan Qi^3^,

Tian-fang Huang^4^, Guo-quan Hu^1^, and Ming-xiong He^1^*

^1^ *Biomass Energy Technology Research Centre, Key Laboratory of Development and Application of Rural Renewable Energy (Ministry of Agriculture and Rural Affairs), Biogas Institute of Ministry of Agriculture and Rural Affairs, Section 4-13, Renmin Rd. South, Chengdu 610041, P. R. China.*

^2^ *Graduate School of Chinese Academy of Agricultural Science, Beijing 100081, P. R. China.*

^3^ *College of Pharmacy and Biological Engineering, Chengdu University, No. 2025, Cheng Luo Road, Chengdu 610106, P. R.China.*

^4^ *Chengdu Institute of Biology, Chinese Academy of Sciences, Section 4-9, Renmin Rd. South, Chengdu 610041, P. R. China.*

^†^*These authors contributed equally to this work.*

*Corresponding author (Fax: +86-28-85242281; E-mail: [hemingxiong@caas.cn](mailto:hemingxiong@caas.cn))


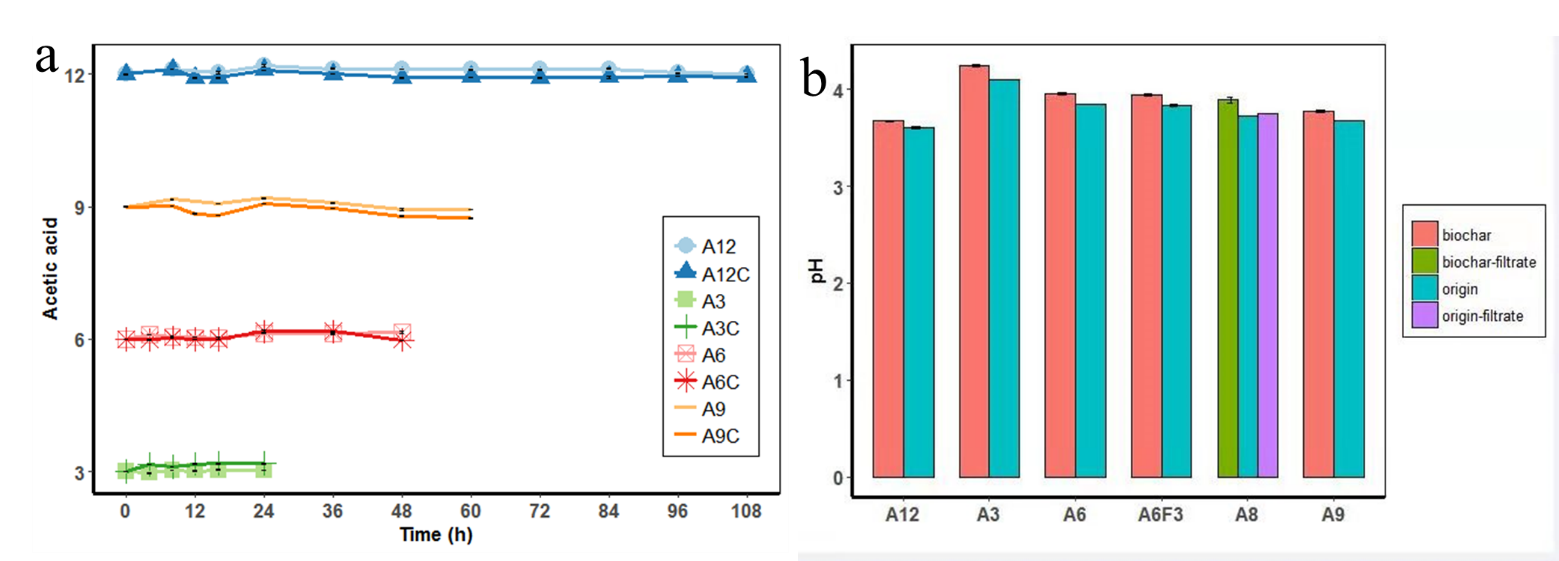
**Figure S1**

Biochar-facilitated ethanol fermentation in acetic acid stress conditions. No acetic acid removal (a), and little pH changes (b)

**Figure S2**

**
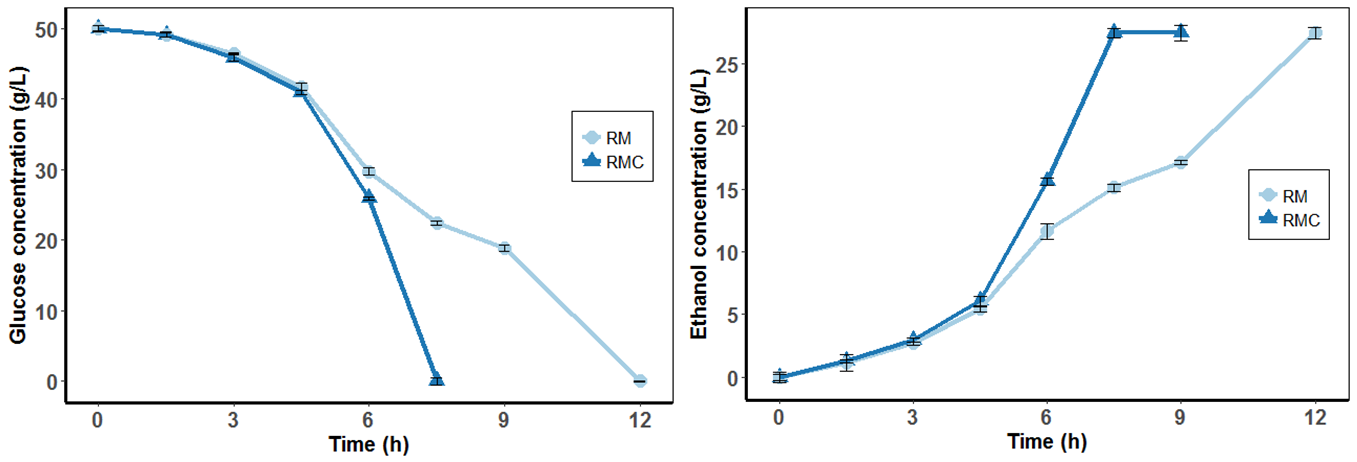
**

Effects of biochar on ethanol fermentation by *Z. mobilis* ZM4 in the presence of RM medium

“RM” indicates *Z. mobilis* ZM4 fermented in RM medium. “RMC” indicates *Z. mobilis*

ZM4 co-cultured with 3.5‰ biochar fermented in RM medium.
